# Severe coronavirus disease 2019 (COVID‐19) pneumonia patients treated successfully with a combination of lopinavir/ritonavir plus favipiravir: Case series

**DOI:** 10.1002/ccr3.3358

**Published:** 2020-09-25

**Authors:** Hayato Koba, Taro Yoneda, Tomoya Kaneda, Tsukasa Ueda, Hideharu Kimura, Kazuo Kasahara

**Affiliations:** ^1^ Respiratory Medicine Komatsu Municipal Hospital Komatsu city Japan; ^2^ Cellular Transplantation Biology Kanazawa University Graduate School of Medical Science Kanazawa city Japan; ^3^ Cardiovascular medicine Komatsu Municipal Hospital Komatsu city Japan; ^4^ Respiratory Medicine Kanazawa University Hospital Kanazawa city Japan

**Keywords:** COVID‐19, favipiravir, ferritin, Lopinavir, lymphocytopenia, ritonavir

## Abstract

The combination therapy of Lopinavir/Ritonavir plus Favipiravir might be a treatment option for patients with COVID‐19. Serum ferritin levels and lymphocytopenia are promising markers for disease severity and disease progression that are commonly available in general clinical practice.

## Graphical abstract

This is the first report to present a promising anti‐viral combination therapy, Lopinavir/Ritonavir plus Favipiravir, for COVID‐19 patients. Serum ferritin levels and lymphocytopenia might be markers for disease severity and disease progression.

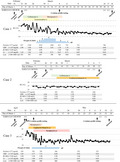

## 1 INTRODUCTION

Three COVID‐19 patients who were received lopinavir/ritonavir plus favipiravir got to improved without any severe adverse events. Two patients harboring high fever, severe pneumonia and respiratory failure obtained dramatic improvement. The combination therapy might be a treatment option; earlier therapy onset may have needed to avoid lung sequela.

While coronavirus disease 2019 (COVID‐19) has spread rapidly worldwide, evidence on effective treatments for it is scant. To the best of our knowledge, we describe here the first report on patients undergoing a successful treatment consisting of a combination of antiviral agents that included favipiravir.

Informed consents from the patients were obtained, and a written consent for publication in this journal was also obtained from each patient. The ethics committee of the Komatsu Municipal Hospital approved the off‐label use of both lopinavir/ritonavir and favipiravir before the administration of each drug.

## 2 CASE REPORT

### 2.1 Case 1

**FIGURE 1:**
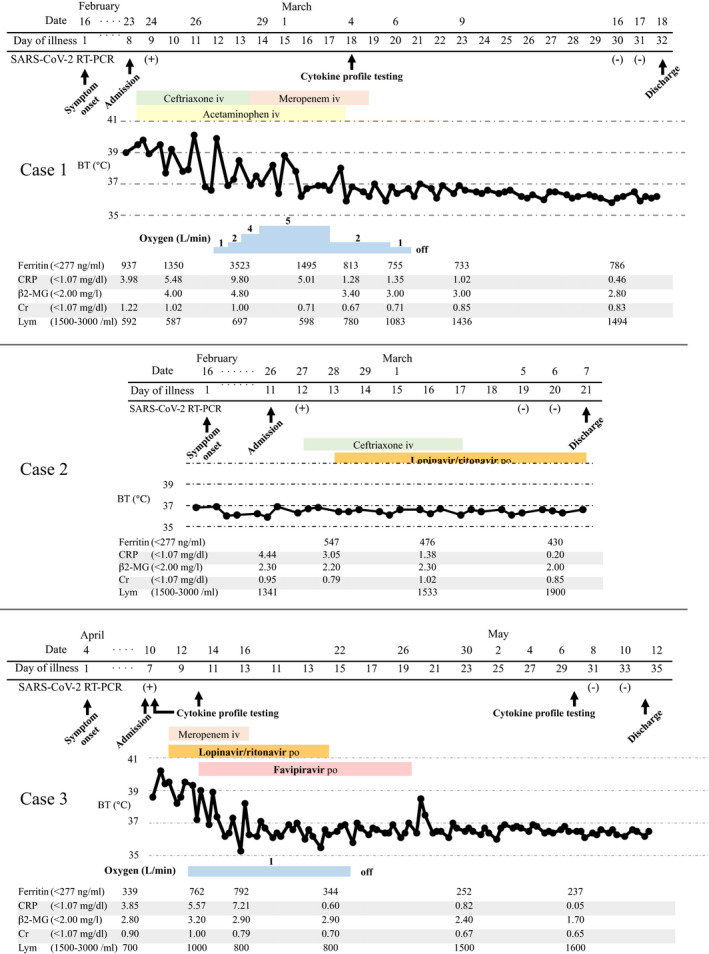
Timeline of the patients' clinical courses from the time of admission. Abbreviation: COVID‐19, coronavirus disease 2019; CT, computed tomography; iv, intravenous; po, orally; BT, body temperature; SARS‐CoV‐2, severe acute respiratory syndrome coronavirus 2; RT‐PCR, real‐time reverse transcriptase polymerase chain reaction; CRP, C‐reactive protein; β2‐MG, β2‐microglobulin; Cr, creatinine; Lym; lymphocyte

**FIGURE 2:**
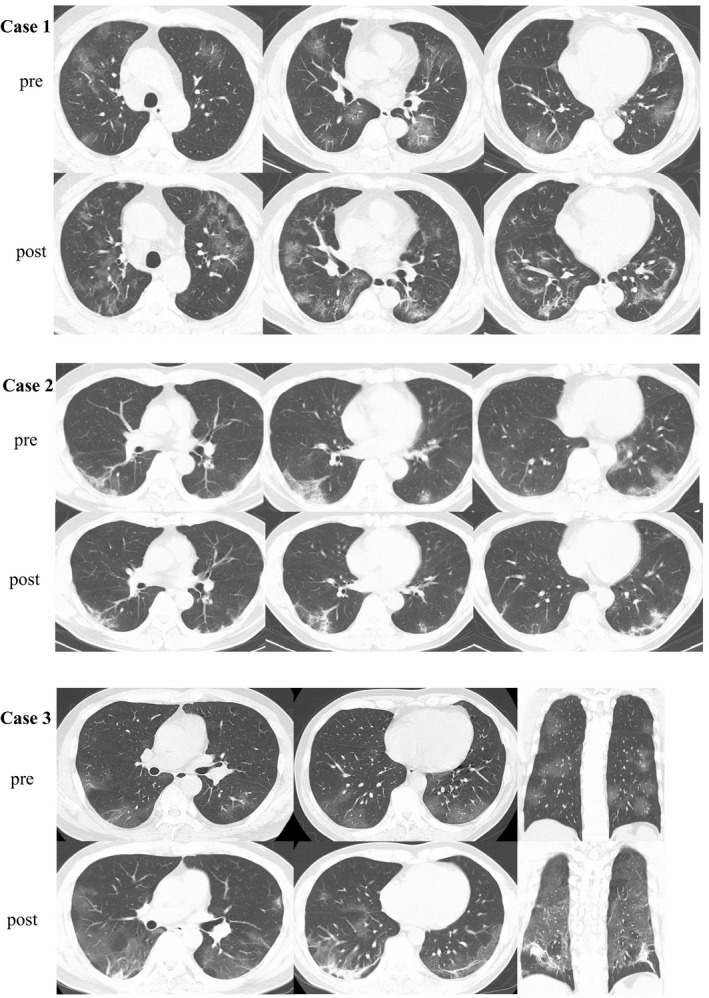
Comparison of CT findings between pre‐ and post‐treatment for COVID‐19 pneumonia. In case 1, the post‐treatment chest CT revealed that GGO in bilateral lungs were increased, furthermore intra‐ and interlobular septal thickening region, bronchiectasis within GGO were emerged. In case 2, the chest CT images reveal bilateral small ground‐glass opacities with linear shadows located dorsally in the lower lung lobes. Post‐treatment chest CT revealed while a part of GGO regions were improved improvement, the linear shadow in GGO was thickened. In case 3, bilateral GGO and septal thickening region were progressed. The regions were predominantly in dorsal. Abbreviation: CT, computed tomography; COVID‐19, coronavirus disease 2019; GGO, ground‐glass opacities

A 61‐year‐old Japanese man was admitted to our hospital on 23 February 2020 with fever, general malaise, and diarrhea. He had a history of recent travel from Japan to Europe on 9 February and returned to Japan on 15 February. On 16 February, (day 1 of illness) he reported general malaise and a fever of less than 38°C. Because of continued fever and malaise and the onset of diarrhea, he visited our hospital on 23 February (day 8 of illness). Upon admission, his body temperature was 39.7°C, dyspnea was not observed, and his oxygen saturation was 99% on ambient air. Laboratory test results showed lymphocytopenia (592/μL) and elevated C‐reactive protein (CRP 3.98 mg/dL) and ferritin (937 ng/mL) levels (Figure 1). Chest radiography and chest computed tomography (CT) revealed multiple bilateral ground‐glass opacities (GGO) in his lungs (Figure 2). The day after admission (day 9 of illness), his nasal swab sample was positive for COVID‐19 by a real‐time reverse transcriptase (RT) polymerase chain reaction (PCR) assay for severe acute respiratory syndrome coronavirus 2 (SARS‐CoV‐2).

Figure 1 shows the patient's clinical course. He was immediately admitted to the isolation ward. Regardless of immediate administration of empiric antibiotic therapy (ceftriaxone [2 g daily]) and antipyretic medication, his hyperpyrexia did not improve and his hypoxemia gradually worsened. On 26 February (day 11 of illness), oxygen administration was initiated because of the respiratory failure, and antiviral therapy with lopinavir/ritonavir (400 mg/100 mg twice daily) was started. However, his respiratory failure got worsened. On 29 February (day 14 of illness), favipiravir (1800 mg twice daily on day 1; 800 mg twice daily, days 2‐14) was added to the lopinavir/ritonavir therapy. Two days after the addition of favipiravir (day 16 of illness), his hyperpnea, pyrexia, appetite, and general malaise were clearly improved. On 6 March (day 20 of illness), the patient no longer required oxygen. The patient's course as reflected by the laboratory data showed that while the CRP and ferritin levels gradually increased after admission, those levels gradually decreased and the patient's lymphocytopenia gradually improved after the initiation of lopinavir/ritonavir and the addition of favipiravir. Representative cytokine levels were examined on 4 March (day 18 of illness); interleukin (IL)‐5 (4 pg/mL), IL‐6 (10 pg/mL), tumor necrosing factor α (TNF‐α) (4.6 pg/mL), and interferon γ (IFN‐) (7.8 pg/mL) (Table 1). After twice confirmation of negative for RT‐PCR for SARS‐CoV‐2, he was discharged on 18 March (day 32 of illness). The post‐treatment chest CT revealed that GGO in bilateral lungs were increased, and however, intra‐ and interlobular septal thickening region, bronchiectasis within GGO were emerged on 17 March (Figure 2). Obvious toxicity related to the combination therapy was never observed.

**TABLE 1:** Cytokine profile

		Case 1	Case 3		
		Mar. 4	Apr. 10	Apr. 13	May. 7
IL‐1β	(−pg/mL)	0.2	ne	ne	ne
IL‐5	(<4 pg/mL)	<4	ne	ne	ne
IL‐6	(<8 pg/mL)	10	32	41	0.9
IL‐8	(<8 pg/mL)	9	ne	ne	ne
TNF‐α	(<1.1 pg/mL)	4.6	0.9	1.5	2.4
IFN‐γ	(<7.8 pg/mL)	<7.8	<7.8	8.3	<7.8
TGFβ1	(<3240 pg/mL)	ne	1978	3817	5991
IL‐10	(<8 pg/mL)	<8	ne	ne	ne
IL‐18	(−pg/mL)	352	ne	ne	ne

Abbreviations: IFN, interferon; IL, interleukin; ne, not evaluable; TGF, tumor growth factor; TNF, tumor necrosing factor.

### 2.2 Case 2

A 50‐year‐old Japanese man was admitted to our hospital on 26 February 2020 with fever, nausea, and cough. His travel history was similar to that of case 1. He reported that he first developed a fever of 38.5°C on 16 February (day 1 of illness). His initial laboratory test results showed an elevated CRP (4.44 mg/dL) level. Chest radiography revealed very small ground‐glass opacities in the lower right lung and a linear shadow in the lower left lung. Chest CT revealed that the small amount of ground‐glass opacities with linear shadows were located dorsally and bilaterally in the lower lobes of the lungs (Figure 1). COVID‐19 was confirmed from a nasal swab specimen assayed for SARS‐CoV‐2 by real‐time RT‐PCR.

Figure 2 shows the patient's clinical course. Empiric antibiotic therapy (ceftriaxone [2 g daily]) was first administered. On 28 February (day 13 of illness), lopinavir/ritonavir (400 mg/100 mg twice daily) was started. On 29 February (day 14 of illness), favipiravir (1800 mg twice daily on day 1; 800 mg twice daily, days 2‐7) was added. Throughout the patient's hospitalization, he remained afebrile and asymptomatic. Twice RT‐PCR assay for SARS‐CoV‐2 was negative, and he was discharged from the hospital on 7 March (day 22 of illness). The patient's course as reflected by the laboratory data showed that his CRP levels gradually decreased after admission. However, his ferritin level worsened once and gradually improved after starting lopinavir/ritonavir and adding favipiravir. Post‐treatment chest CT revealed while a part of GGO regions were improved improvement, the linear shadow in GGO was thickened 17 March (Figure 2). Obvious toxicity related to the combination antiviral therapy was never observed.

### 2.3 Case 3

A 54‐year‐old Japanese man was admitted to our hospital on 10 April 2020 with fever, dyspnea, and diarrhea. He had been Tokyo from 30 to 31 March to help his son with house moving. He reported that he first developed a fever of 39°C on 4 April (day 1 of illness). His initial laboratory test results showed an elevated CRP (3.85 mg/dL) level. Chest CT revealed diffuse ground‐glass opacities in bilateral lungs (Figure 1). COVID‐19 was confirmed from a nasal swab specimen assayed for SARS‐CoV‐2 by real‐time RT‐PCR.

Figure 2 shows the patient's clinical course. Although lopinavir/ritonavir (400 mg/100 mg twice daily) and meropenem (1 g thrice daily) was started on 11 April (day 8 of illness), he got to worse as a respiratory failure on the next day. On 13 April (day 10 of illness), favipiravir (1800 mg twice daily on day 1; 800 mg twice daily, days 2‐14) was added. Four days after the addition of favipiravir (day 16 of illness), his hyperpnea, pyrexia, appetite, and general malaise were improved. On 22 April (day 19 of illness), the patient no longer required oxygen. The patient's course as reflected by the laboratory data showed that while the CRP, ferritin, and β2 microglobulin levels gradually increased after admission, those levels gradually decreased and the patient's lymphocytopenia gradually improved after the addition of favipiravir. Representative cytokine levels were examined on 10, 13 April, and 7 May (day 7, 10, and 30 of illness, respectively). IL‐6, TNF‐α and tumor growth factor β1(TGFβ1) level were abnormally elevated (Table 1). After twice confirmation of negative for RT‐PCR for SARS‐CoV‐2, he was discharged on 11 May (day 34 of illness). The post‐treatment chest CT revealed that bilateral intra‐ and interlobular septal thickening region were emerged on 11 May (Figure 2). Obvious toxicity related to the combination therapy was never observed.

## 3 DISCUSSIONS

To the best of our knowledge, this is the first report indicating that combination antiviral therapy consisting of lopinavir/ritonavir and favipiravir might prove beneficial for patients with COVID‐19. These agents relieved life‐threatening lung injury in cases 1, 3, and in the other case provided early negative RT‐PCR for SARS‐CoV‐2 results from a nasal swab specimen on day 7 after the initiation of antiviral therapy.

Although approximately 80% of COVID‐19 patients have been reported to have mild disease,^1^, ^2^ the mortality rate of COVID‐19 has so far been reported to range from 1.4% to 15%^1^, ^2^, ^3^; and the case‐fatality rate of patients classified as critical, such as case 1, has been reported to be 49.0%.^1^ While the causes of death from these reports^2^, ^3^ were unclear, the acute respiratory distress syndrome might have been related to disease severity. The histopathological features of COVID‐19‐induced lung injury resemble those of the severe acute respiratory syndrome (SARS) and middle eastern respiratory syndrome.^4^ A higher incidence of physician‐diagnosed pneumonia was observed in patients with severe disease than in those with less severe disease, and 3.4% of all COVID‐19 patients progressed to acute respiratory distress syndrome afterward.^2^ Thus, the establishment of predictive markers for the degree of disease severity, followed by the appropriate treatment for severely ill patients, are urgent priorities. We think that this report might be valuable with regard to these issues (predictive markers and life‐saving treatment).

The protease inhibitor lopinavir/ritonavir, which is approved for the treatment of human immunodeficiency virus‐1 infection, was reported to be effective for five patients with COVID‐19 in Singapore. They all were cured.^5^ However, a randomized controlled trial to verify whether severe COVID‐19 patients get clinical improvement to receive lopinavir/ritonavir in China reported no benefit.^6^ Favipiravir, which is being stockpiled for use as a countermeasure for novel influenza, functions as a chain terminator at the site of the incorporation of viral RNA and reduces the viral load. The agent resembles a nucleoside analogue, functioning as a purine homologue, and therefore inhibits viral RNA synthesis.^7^ It has broad‐spectrum activity against RNA viruses such as the Ebola virus, Lassa virus, rabies, and the virus that causes severe fever with thrombocytopenia syndrome.^7^, ^8^ An open‐label nonrandomized control study reported COVID‐19 patients treated by favipiravir showed significantly higher improvement in chest imaging and faster viral clearance than those treated by lopinavir/ritonavir.^9^

Commonly reported adverse reactions to lopinavir/ritonavir have included diarrhea, nausea, vomiting, hypertriglyceridemia, and hypercholesterolemia.^10^ The adverse reactions to favipiravir include teratogenicity, increased blood levels of uric acid, diarrhea, and neutropenia. Hypertriglyceridemia and hypercholesterolemia were observed in case 1. Although there are no safety data on the simultaneous use of lopinavir/ritonavir plus favipiravir, based on the kinetic mechanisms of the agents, the drug‐drug interactions appear to be minimal.^10^ Immediately after termination of favipiravir medication in case 3, transient febrile reaction was observed. Although it is not easy to distinguish infectious fever from iatrogenic drug fever, it makes us suggest the importance to remember the chemical fever even in antiviral therapy.

The progression of COVID‐19 seems to be associated with a ‘cytokine storm’, which is also true of SARS and Middle Eastern Respiratory Syndrome. Elevated ferritin levels and lymphocytopenia seemed to be an accurate reflection of the severity of COVID‐19 in our 3 patients, based on their clinical courses (Figure 1). COVID‐19 patients who needed admission to intensive care unit had less lymphocyte counts than counterpart.^3^ Our findings suggest that IL‐6 and TNF‐α which were reflected by the serum CRP and ferritin level, have a central role in the progression of COVID‐19, as opposed to IFN‐γ (Table 1). Chaolin H et al^3^ reported that a patient with severe COVID‐19 had high concentrations of GCSF, IP10, MCP1, MIP1A, and TNF‐α, which led to activation of T‐helper 1 cells. On the other hand, they also reported increased secretion of T‐helper 2 cytokines.^3^ The ferritin, CRP, and β2‐microglobulin levels decreased in cases 1 and 3 after the administration of favipiravir. These findings suggest that favipiravir not only reduces the levels of inflammatory cytokines in vitro, but might also reduce the levels in vivo.^7^ Furthermore, earlier therapy onset may have needed to avoid lung sequela now that chest CT findings at post‐treatment suggest remaining pulmonary fibrosis (Figure 2). According to the radiological study,^11^, ^12^ COVID‐19 must leave pulmonary scars like SARS.^13^, ^14^

This case series has limitations. First, we described only three patients; it was very difficult to generalize these findings. Second, they may have improved without any antiviral therapy. Third, the unavoidable delay to the diagnosis of COVID‐19 impedes the ability to determine when we should initiate antiviral therapy.

In conclusion, the combination therapy of lopinavir/ritonavir plus favipiravir might be a treatment option for patients with COVID‐19. Serum ferritin levels and lymphocytopenia are promising markers for disease severity and disease progression that are commonly available in general clinical practice. Prospective study in subjects with large number of COVID‐19 patients is needed to clarify these effectiveness.

## CONFLICT OF INTEREST

The authors have reported to Respiration that no potential conflicts of interest exist with any companies/organizations whose products or services may be discussed in the article.

## AUTHOR CONTRIBUTIONS

HK: involved in conceptualization, investigation, writing‐original draft, visualization. TY: involved in data curation and investigated. TK and TU: investigated. HK: wrote, reviewed, and edited the manuscript and visualized. KK: supervised. Both authors have read and approved the final manuscript.

## ETHICAL APPROVAL

Informed consent from the patient was obtained, and a written consent for publication in this journal was also obtained from each patient. The ethics committee of the Komatsu Municipal Hospital approved the off‐label use of both lopinavir/ritonavir and favipiravir before the administration of each drug, and the presentation or publication about these cases. Notation of prior abstract/presentation: This paper of any similar paper has not been submitted nor published in any other journal.
